# Body Mass Index (BMI) Is Associated with Microalbuminuria in Chinese Hypertensive Patients

**DOI:** 10.3390/ijerph120201998

**Published:** 2015-02-10

**Authors:** Xinyu Liu, Yu Liu, Youming Chen, Yongqiang Li, Xiaofei Shao, Yan Liang, Bin Li, Harry Holthöfer, Guanjing Zhang, Hequn Zou

**Affiliations:** 1 Institute of Nephrology and Urology, The Third Affiliated Hospital of Southern Medical University, Guangzhou 510630, China; E-Mails: xinyuliu2014@hotmail.com (X.L.); pepsi84@163.com (Y.L.); shaoxfei@126.com (X.S.); lsky2008@yeah.net (Y.L.); libin2014@163.com (B.L.); 2 Division of Birth Cohort Study, Guangzhou Women and Children’s Medical Center, Guangzhou 510623, China; E-Mail: mzmxly81@163.com; 3 Department of Statistics, School of Mathematics and Computational Science, Sun Yat-Sen University, Guangzhou 510275, China; 4 Clinical Laboratory Centre, The Third Affiliated Hospital of Southern Medical University, Guangzhou 510630, China; E-Mail: cym38432@163.com; 5 Centre for Bio Analytical Sciences (CBAS), Dublin City University, Dublin 9, Ireland; E-Mail: harry.holthofer@dcu.ie; 6 E-Techco Information Technologies Co., Ltd., Shenzhen 518057, China; E-Mail: john.zhang@e-techco.com

**Keywords:** hypertension, mircroalbuminuria, BMI

## Abstract

There is no general consensus on possible factors associated with microalbuminuria in hypertensive patients nor any reported study about this issue in Chinese patients. To examine this issues, 944 hypertensive patients were enrolled in a study based on a cross-sectional survey conducted in Southern China. Multivariate regression analyses were performed to identify the factors related with the presence of microalbuminuria and urinary excretion of albumin. The prevalence of microalbuminuria in hypertensive and non-diabetic hypertensive patients were 17.16% and 15.25%, respectively. Body mass index (BMI), but not waist circumference (WC), were independently associated with microalbuminuria and the values of urinary albumin to creatinine ratio (ACR) based on multiple regression analyses, even after excluding diabetic patients and patients taking inhibitors of the renin-angiotensin system from the analyses. Furthermore, patients with obesity (BMI ≥28) had higher levels of ACR, compared with those with normal weight (BMI <24 kg/m^2^) and overweight (24 kg/m^2^≤ BMI < 28). In conclusion, BMI, as a modifiable factor, is closely associated with microalbuminuria among Chinese hypertensive patients, which may provide a basis for future development of intervention approaches for these patients.

## 1. Introduction

Microalbuminuria refers to a clinical abnormality with a moderately increased amount of albumin extracted to the urine, presumably due to glomerular capillary damage [1]. It is widely accepted that microalbuminuria is associated with a widespread vascular dysfunction and is considered as an indicator of increased cardiovascular risk and all-cause mortality, especially for high-risk populations such as hypertensive patients [2,3]. Several guidelines recommend a regular screening for microalbuminuria in hypertensive patients [4,5]. Intervention measures against the occurrence or progression of this early stage renal injury in hypertensive patients can delay or prevent end-stage kidney disease (ESRD) and severe cardiovascular diseases. Therefore, characterization of possible risk factors for the presence of microalbuminuria in hypertensive patients may provide valuable information for clinical intervention.

Although hypertension is considered the second leading cause of ESRD worldwide, the risk for patients with uncomplicated essential hypertension to develop serious renal injury is relatively low [5]. This indicates that there may be coexisting factors that amplify the renal damage from high blood pressure. Several studies have been conducted to identify possible factors related to microalbuminuria and/or the excretion of urinay albumin in hypertensive patients, but the results are inconsistent and sometimes conflicting [6,7,8,9,10,11]. To our best knowledge, there are no such reports on microalbuminuria among hypertensive patients in China, while the prevalence of hypertension and chronic kidney disease (CKD) in China has be increasing dramatically over the past few decades [12,13]. The present study was designed to investigate the prevalence of microalbuminuria and possible associated risk factors in Chinese hypertensive patients using a large sample size. It is hoped that our results would provide a basis for future development of comprehensive intervention approaches to prevent and manage kidney diseases in this specific population.

## 2. Methods

### 2.1. Study Design and Subjects

This study was based on the data obtained from our population-based, cross-sectional survey, which conducted in Wanzai Town, Zhuhai City, Tangxia Town and Guangzhou City in Southern China from June 2012 to February 2013. The study protocol was approved by the Ethics Committee of The Third Affiliated Hospital of Southern Medical University (Guangzhou, China). All adults (age > 18 years) were invited to participate in this survey. Individuals with fever, or signs of acute disease and recent infection, and women who were actively menstruating were excluded from the survey. A total of 2667 subjects participated in this survey (159 refused). All participants provided written informed consent. The details of this survey were described in our previously published paper [14]. In the present study, only the data from the subjects with the diagnosed of hypertension (according to the blood pressure measurement or history of essential hypertension) were included. After further exclusion of subjects diagnosed with secondary hypertension (*n* = 12) or presence of macroalbuminuria (urinary albumin to creatinine ratio (ACR) ≥ 300 mg/g, *n* = 93), or women who were pregnant (*n* = 5), the data of 944 hypertensive patients were analyzed in the present study.

### 2.2. Demographic, Anthropometrical, and Clinical Data Collection

Demographic and clinical information including age, gender, education, current or past smoking, alcohol use, physical activity, and medical and medication history, were obtained by questionnaires in local community clinic or during home interview. Blood pressure (BP) was measured by trained nurses using a calibrated manual mercury sphygmomanometer (Yuyue Medical Instruments Co., Ltd., Jiangsu, China) for all participants in a seated position after resting for 5 min. BP readings were taken three times and the average values of systolic blood pressure (SBP) and diastolic blood pressure (DBP) were recorded. Weight and height were measured in light clothing without shoes, and body mass index (BMI) was calculated as weight in kilograms divided by the square of height in meters. Waist circumference (WC) was measured at a level midway between the lowest rib and the iliac crest.

### 2.3. Laboratory Tests

Blood specimens were collected from participants after overnight fasting for at least 10 h. First void morning urine samples were also collected. Serum insulin was measured using an electrochemiluminescence immunoassay. Urinary creatinine, serum creatinine, serum uric acid (UA), fasting glucose, and serum triglyceride (TG), low density lipoprotein cholesterol (LDL-C), and high density lipoprotein cholesterol (HDL-C) were measured by a colorimetric method. High sensitivity C-reactive protein (hs-CRP) was detected using an enzymatic immunoassay turbidimetric method. Urinary albumin was measured by an immune nephelometric method.

The estimated glomerular filtration rate (eGFR) was calculated by using the four-variable Modification of Diet in Renal Disease (MDRD) equation [175 × (Scr)^−1.234^ × (Age)^−°.179^ × (if female, × 0.79)] [15]. The urinary excretion of albumin was expressed as the ratio of urinary albumin to creatinine (ACR, mg/g). Since the detection limit of albuminuria in our laboratory was 5 mg/L, the value reported as <5 mg/L (8 patients) was considered as 5mg/L for calculating ACR and other analyses.

### 2.4. Definitions

Hypertension was defined as BP ≥ 140/90mmHg or having a confirmed diagnosis of hypertension before this survey. Obesity was defined as BMI ≥ 28 kg/m^2^. Overweight was defined as BMI ≥ 24 kg/m^2^ and <28 kg/m^2^ [16]. Diabetes mellitus was defined as a fasting serum glucose ≥7.0 mmol/L or having a confirmed diagnosis of diabetes. Microalbuminuria was defined as having a ACR value between >30 and ≤299 mg/g [17].

### 2.5. Statistical Analysis

All statistical analyses were performed with SPSS 17.0 software (Chicago, IL, USA). Continuous variables were depicted as Mean ± SD, except for ACR and hs-CRP, which were displayed as median and interquartile range (IQR). For the categorical variables, absolute and relative (%) values were presented. Baseline characteristics of patients with normal albuminuria and microalbuminuria were compared, and Student’s *t* test or Wilcoxon rank-sum test was used for analyses of continuous variables and the chi-squared test was used for categorical variables.

Univariate and multivariate logistic regression analysis (backward stepwise) were performed to determine the association between microalbuminuria (as the dependent variable) and other variables. To avoid the multicollinearity, BMI, and WC were included into different multivariate logistic regression models. The goodness-of-fit of the logistic regression models was assessed by the Hosmer and Lemeshow test. The same logistic regression analyses were conducted after excluding diabetic patients and subjects taking inhibitors of the renin-angiotensin system. Furthermore, univariate and multiple linear regression analyses were performed to examine the relationships between ACR and other variables. One-way ANOVA and subsequent multiple comparisons analysis (SNK *q* test) were used for comparing the ACR levels among subjects with normal weight, overweight and obesity. The ACR and hs-CRP values were normalized by logarithmical transformation for the statistical analyses. Shapiro-Wilk test was used to assess the normality for log(ACR) in the normal weight, overweight, and obesity subpopulations. A two-tailed *p* value <0.05 was considered statistically significant.

## 3. Results

Among the 944 hypertensive subjects (aged 60.79 ± 11.3 years) included in the present study, 37.7% of them were male. One hundred and sixty two (162, 17.16%) subjects had microalbuminuria. Thirty two (32) subjects were taking angiotensin-converting enzyme inhibitors (ACEIs) and 12 subjects were using angiotensin II receptor antagonists (ARBs) before the survey, while 139 subjects were taking other hypertensive drugs, including calcium channel blockers (104 cases), thiazidediuretics (25 cases), and β-blockers (10 cases). The comparative data of patients with normal albuminuria and microalbuminuria are presented in Table 1. Compared with those with normal albuminuria, patients with microalbuminuria were older, had increased values of BMI, waist circumference, SBP, fasting glucose, serum insulin, and hs-CRP. In addition, diabetes is more prevalent in patients with microalbuminuria. There were no significant differences in gender, education attainment, physical activity, smoking, alcohol use, DBP, serum creatinine, eGFR, UA, TG, LDL-C, or HDL-C between the patients with and without microalbuminuria.

As shown in Table 2, univariate analyses indicated thatthe presence of microalbuminuria was positively related to age, BMI, WC, SBP, fasting glucose, insulin, and hs-CRP, respectively. However, only age (1.03 (1.01–1.05), *p* = 0.005), BMI (1.10 (1.04–1.16), *p* < 0.001), SBP (1.02 (1.01–1.04), *p* < 0.001), and fasting glucose (1.26 (1.14–1.39), *p* < 0.001) were independently associated with microalbuminuriain a multivariate logistic regression model (model 1). In another model (model 2) analysis where WC was adopted instead of BMI, microalbuminuria was independently associated with age, SBP, fasting glucose, but not WC. Furthermore, in the analysis excluding patients with diabetes (*n* = 144), 122 (15.25%) of non-diabetic hypertensive patients had microalbuminuria, and age, BMI and SBP were still independently associated with the presence of microalbuminuria, while there was not relationship between microalbuminuria and fasting glucose. In addition, to eliminate the possible effect of renin-angiotensin system (RAS) inhibitors on microalbuminuria, the same multivariate regression analysis was conducted by excluding patients taking these medications (*n* = 44) and WC was, again, not significantly associated with microalbuminuria (data not shown).

**Table 1:** The comparative between subjects with normal albuminuria and microalbuminuria.

Parameter	Normal Albuminuria (*n* = 782)	Microalbuminuria (*n* = 162)	*p*
ACR, mg/g	9.72 (6.72–14.67)	50.83 (36.07–84.25)	<0.001
Male, n (%)	300 (38.36%)	56 (34.57%)	0.364
Age, years	60.22 ± 11.24	63.52 ± 11.24	0.001
Education attainment High school or above, n (%)	209 (26.73%)	35 (21.61%)	0.175
Physical inactivity, n (%)	343 (43.86%)	75 (46.30%)	0.570
Current smoker, n (%)	83 (10.61%)	17 (10.49%)	0.964
Current alcohol use, n (%)	152 (19.44%)	28 (17.28%)	0.525
Diabetes, n (%)	104 (13.30%)	40 (24.69%)	<0.001
BMI, kg/m^2^	24.37 ± 3.32	25.29 ± 4.24	0.013
WC, cm	86.67 ± 9.40	88.90 ± 9.61	0.007
SBP, mmHg	145.60 ± 16.00	151.77 ± 18.31	<0.001
DBP, mmHg	85.41 ± 10.46	86.05 ± 11.26	0.451
Fasting glucose, mmo/L	5.15 ± 1.25	6.01 ± 2.60	<0.001
Serum insulin, nU/L	11.33 ± 7.38	13.08 ± 9.33	0.041
Serum creatitine, umol/L	75.58 ± 16.45	74.27 ± 17.82	0.467
eGFR, mL/min/1.73m^2^	93.09 ± 20.04	94.15 ± 22.28	0.495
UA, umol/L	368.98 ± 96.71	370.50 ± 107.22	0.866
hs-CRP, mg/L	1.33 (0.64–2.90)	1.94 (0.89–4.01)	0.027
TG, mmol/L	1.66 ± 0.95	1.84 ± 1.07	0.222
LDL-C, mmol/L	3.30 ± 0.91	3.24 ± 0.86	0.698
HDL-C, mmol/L	1.51 ± 0.32	1.51 ± 0.31	0.926

Mean ± SD or median (interquartile range) for continuous variables and % for categorical variables are presented. Abbreviations: ACR, albumin to creatinine ratio; BMI, body mass index; WC, waist circumference; SBP, systolic blood pressure; DBP, diastolic blood pressure; eGFR, estimated glomerular filtration rate; UA, uric acid ; hs-CRP, high sensitivity C-reactive protein; TG, triglyceride; LDL-C, low-density lipoprotein cholesterol; HDL-C, high-density lipoprotein cholesterol.

In the following series of analyses, ACR was taken as a continuous variable. Table 3 presents the results of univariate and multivariate linear regression analysis for the association between ACR and other variables. Univariate analysis demonstrated that age, SBP, and fasting glucose were positively correlated with ACR, while gender showed an inverse correlation. Then backward stepwise method was applied in order to select variables included in multivariate regression model 1 (including BMI) and model 2 (including WC). Multivariate regression analysis revealed that gender, age, SBP, fasting glucose, BMI, but not WC, were the independently associated withACR (Table 3). Similar results were obtained in regression analyses of a subgroup of patients without diabetes and another subgroup of patients without taking RAS inhibitors.

**Table 2:** Univariate and multivariate logistic regression analyses for the association between microalbuminuria and various variables in hypertensive patients.

Variables	Univariate		Multivariate			
	Odds Ratio (95% CI)	*p*	Model 1(BMI)		Model 2(WC)	
			Odds Ratio (95% CI)	*p*	Odds Ratio (95% CI)	*p*
Gender, male	0.85 (0.60–1.21)	0.375	—	—	—	—
Age, years	1.21 (1.01–1.04)	0.001	1.03 (1.01–1.05)	0.005	1.03 (1.02–1.05)	0.007
Physical activity	0.94 (0.67–1.33)	0.741	—	—	—	—
Smoke statues	0.95 (0.72–1.24)	0.687	—	—	—	—
Alcohol use	0.95 (0.77–1.19)	0.67	—	—	—	—
BMI, kg/m^2^	1.07 (1.02–1.12)	0.004	1.10 (1.04–1.16)	<0.001	—	—
WC, cm	1.03 (1.01–1.05)	0.008	—	—	—	—
SBP, mmHg	1.02 (1.01–1.03)	<0.001	1.02 (1.01–1.04)	<0.001	1.02 (1.01–1.03)	<0.001
DBP, mmHg	1.01 (0.99–1.02)	0.451	—	—	—	—
Fasting glucose, mmo/L	1.28 (1.17–1.40)	<0.001	1.27 (1.15–1.39)	<0.001	1.29 (1.18–1.42)	<0.001
Insulin, nU/L	1.02 (1.00–1.04)	0.021	—	—	—	—
Serum creatinine, umol/L	1.00 (0.99–1.01)	0.494	—	—	—	—
UA, umol/L	1.00 (0.99–1.00)	0.866	—	—	—	—
hs-CRP, mg/L	1.04 (1.00–1.07)	0.029	—	—	—	—
TG, mmol/L	1.08 (0.95–1.22)	0.225	—	—	—	—
LDL-C, mmol/L	0.96 (0.80–1.16)	0.698	—	—	—	—
HDL-C,mmol/L	0.98 (0.58–1.64)	0.926	—	—	—	—

Independent variables in the multivariate model were chosen using a backward stepwise regression analysis where all variables listed in the univariate analysis. BMI and WC were selected into model 1 and model 2 separately. Serum insulin was excluded from the multivariate model due to possible multicollinearity. The goodness-of-fit of the logistic regression models was assessed by the Hosmer and Lemeshow test, and the *p* value was 0.525, and 0.277 for model 1 and model 2, respectively. Abbreviations: ACR, albumin to creatinine ratio; BMI, body mass index; WC, waist circumference; SBP, systolic blood pressure; DBP, diastolic blood pressure; eGFR, estimated glomerular filtration rate; UA, uric acid ; hs-CRP, high sensitivity C-reactive protein; TG, triglyceride; LDL-C, low-density lipoprotein cholesterol; HDL-C, high-density lipoprotein cholesterol.

**Figure 1:**
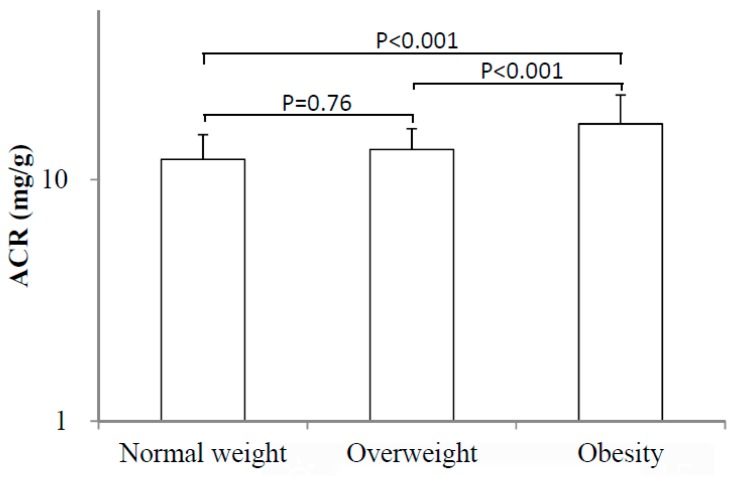
Comparision of ACR values among hypertensive patients with normal weight (BMI < 24kg/m^2^), overweight (24 kg/m^2^ ≤ BMI < 28 kg/m^2^) and obesity(BMI ≥ 28 kg/m^2^).

Based on the above robust results that BMI was independently related with microalbuminuria, as well as ACR in hypertensive patients, we further compared the ACR levels among subjects with normal weight, overweight and obesity. The one-way ANOVA and consequent multiple comparisons analysis revealed that ACR level in obese individuals (15.34 (9.44–30.67)) were significantly higher than that with normal weight (10.70 (7.36–20.00)) and overweight (10.87 (8.81–20.16)) (*p* < 0.001 for both), while no difference was observed between overweight and normal weight subjects (*p* = 0.76) (Figure 1).

**Table 3:** Univariate and multivariate linear regression analysis for correlation between ACR and other variables in hypertensive subjects.

Variables	Univariate		Multivariate					
	*r*	*p*	Model 1(BMI)		*p*	Model 2(WC)		*p*
			B (95% CI)	*t*		B (95% CI)	*t*	
Gender, male	0.157	<0.001	−0.12 (−0.17–−0.07)	−4.75	<0.001	−0.12 (−0.17–−0.07)	−4.64	<0.001
Age, years	0.147	<0.001	0.00 (0.00–0.01)	3.38	0.001	0.01 (0.00–0.01)	3.67	<0.001
Physical activity	0.007	0.829	—	—	—	—	—	—
Smoke statues	0.038	0.249	—	—	—	—	—	—
Alcohol use	0.058	0.077	—	—	—	—	—	—
BMI, kg/m^2^	0.133	<0.001	0.02 (0.01–0.02)	4.20	<0.001	—	—	—
WC, cm	0.081	0.009	—	—	—	—	—	—
SBP, mmHg	0.132	<0.001	0.00 (0.00–0.01)	4.75	<0.001	0.00 (0.00–0.01)	4.56	<0.001
DBP, mmHg	0.001	0.972	—	—	—	—	—	—
Fasting glucose, mmo/L	0.191	<0.001	0.04 (0.03–0.06)	5.66	<0.001	0.04 (0.03–0.06)	5.33	<0.001
Insulin, nU/L	0.08	0.014	—	—	—	—	—	—
Serum creatinine, umol/L	0.022	0.505	—	—	—	—	—	—
UA, umol/L	0.013	0.688	—	—	—	—	—	—
hs-CRP, mg/L	0.085	0.009	—	—	—	—	—	—
TG, mmol/L	0.049	0.13	—	—	—	—	—	—
LDL-C, mmol/L	0.027	0.413	—	—	—	—	—	—
HDL-C, mmol/L	0.011	0.729	—	—	—	—	—	—

Independent variables in the multivariate model were chosen using a backward stepwise regression analysis where all variables listed in the univariate analysis except insulin. BMI and WC were selected into model 1 and model 2 separately. Adjust R2 was 0.14 for model 1 and 0.15 for model 2, and *p*<0.001 for both; Abbreviations: ACR, albumin to creatinine ratio; BMI, body mass index; WC, waist circumference; SBP, systolic blood pressure; DBP, diastolic blood pressure; eGFR, estimated glomerular filtration rate; UA, uric acid; hs-CRP, high sensitivity C-reactive protein; TG, triglyceride; LDL-C, low-density lipoprotein cholesterol; HDL-C, high-density lipoprotein cholesterol.

## 4. Discussion and Conclusions

The prevalence rates of microalbuminuria in hypertensive subjects have been reported to range from 6.6% to 46% [6,10,18,19]. A broad-based international survey indicates that this figure could be as high as 53%–71% in various countries [20]. To our best knowledge, the present cross-sectional study was the first report from China focusing on microalbuminuria in patients with hypertension. We found that the prevalence rates of microalbuminuria in hypertensive and non-diabetic hypertensive patients were 17.16% and 15.25%, respectively, which were similar to the previous reports.

The main purpose of this study was to investigate the potential risk factors associated with microalbuminuria in hypertensive patients, focusing on BMI and WC, with the understanding that these are modifiable risk factors. Our results indicated that age, SBP, and BMI were independently correlated with microalbuminura. Such a relationship remained after excluding subjects with diabetes and those taking inhibitors of the renin-angiotensin system. Moreover, age, SBP, and BMI were also independently correlated with increased ACR. In addition, subjects with obesity (BMI ≥ 28 kg/m^2^) showed significantly higher level of ACR than their counterparts with normal weight and overweight. Therefore, we conclude that BMI, but not WC, is associated with microalbuminuria in Chinese hypertensive patients.

BMI (height-independent measure of weight) and WC (marker of abdominal excess fat) are both to evaluate the excess mass of body fat. Obesity with very high BMI indicates an increase in total body fat and has been shown to be associated with comorbidities such as hypertension, diabetes, and atherosclerotic cardiovascular diseases, as well as total mortality [21].

There are conflicting findings on the association between microalbuminuria and BMI and WC in previous studies. Similar to our study, the data from across-sectional assay conducted in Asian countries have demonstrated that increased BMI was an independent predictor of elevated ACR (47% higher for BMI ≥ 25kg·m^2^) in hypertensive patients [10]. In contrast, the i-SEARCH study which enrolled 20,828 hypertensive patients from 26 countries (mainly from western countries) concluded that abdominal obesity with increased WC, but not BMI, appeared to be associated with microabluminuria [11].

There is accumulating evidence supporting that obesity significantly increases the risk of micoalbuminuria in hypertensive patients [10,11,22]. Hypertension associated microalbuminuria are usually explained by the theory that elevated systemic arterial BP transmits a higher pressure to the glomerular capillaries [22]. It’s widely postulated that obesity is associated with increased glomerular hyperfiltration and hypertrophy, which thus increases the risk of hypertensive glomerular injury due to an enhanced glomerular transmission of systemic pressures and susceptibility of glomerular capillary to barotraumas [23].

Abdominal obesity, or increased WC, is usually considered as a key component of metabolic syndrome (MS). It has been proven that MS could cause endothelial dysfunction of kidneys and glomerular lesions by multiple complex pathogenesis, resulting in microalbuminuria [24,25]. The findings of the present study indicated that abnormal WC alone, without combing with other components of MS, might not be independently correlated with microalbuminuria in Chinese hypertensive patients. A possible explanation for this result might be that our study didn’t involve many subjects with abdominal obesity, which may be linked to the selection of study population. It is generally believed that the individuals with abdominal obesity in China are from the populations with a higher income and living in Western lifestyle. The present study was acommunity-based survey that may not have enrolled such subjects with abdominal obesity.

Of note, the current study had several limitations. First, in spite of having adjusted for confounding factors, the cross-sectional nature of our study does not permit the assessment of an absolute cause-and-effect relationship between microalbuminuria and tested risk factors. To establish such a causal relationship, longitudinal studies in various populations are needed. Second, ACR was obtained on the basis of single measurement; repeated testing may increase the validity of the results. Third, only 37.7% of the subjects were men, the sample might be biased even though gender as a factor was adjusted in multiple regression analysis. Finally, the lifestyle factors were not be well characterized in the present study. It is reported that diet quality *per se* could affect microalbuminuria risk independently [26]. Future studies should examine these lifestyle factors in relation to microalbuminuria and CKD.

Nevertheless, obesity with very high BMI is a modifiable factor and has become a major public health care challenge worldwide and particularly in China. Based on the findings from our study, together with other earlier reports, we suggest that reducing body weight with maintaining healthy lifestyle should be recommended to hypertensive patients to prevent kidney diseases.
